# Poly(Vinyl Alcohol) Drug and PVA–Drug–Surfactant Complex Organogel with Dimethyl Sulfoxide as a Drug Delivery System

**DOI:** 10.3390/gels10110753

**Published:** 2024-11-20

**Authors:** Sabina Otarbayeva, Dmitriy Berillo

**Affiliations:** 1 Department of Chemistry and Biochemical Engineering, Satbayev University, Almaty 050013, Kazakhstan; 2 Department of Biochemistry, Asfendiyarov Kazakh National Medical University, Almaty 050000, Kazakhstan

**Keywords:** antibiotics, drug delivery system, organogel, hydrogel, antimicrobial activity, kinetic of release, polyelectrolyte complex, PVA, DMSO

## Abstract

The relevance of active research lies in the need to develop new technologies to improve drug delivery methods for the effective treatment of wound healing. Additionally, the potential application of organogels in other areas of biomedicine, such as creating medical patches with controlled drug delivery, indicates a wide range of possibilities for using this technology. This study focuses on developing controlled drug delivery systems using organogels as carriers for ceftriaxone and ofloxacin. By selecting optimal formulations, organogels were created to immobilize the drugs, facilitating their effective and sustained release. The swelling behavior of the hydrogels was studied, showing a swelling coefficient between 16 and 32%, indicating their ability to absorb liquid relative to their weight. Drug release studies demonstrated that ceftriaxone was released 1.8 times slower than ofloxacin, ensuring a more controlled delivery. Microbiological tests confirmed that the organogels containing ofloxacin exhibited antimicrobial activity against Escherichia coli, Bacillus subtilis, and Staphylococcus aureus. However, it was a challenge to estimate activity for the model antibiotic ceftriaxone due to bacterial resistance to it. Organogel poly(vinyl alcohol) (PVA)-DMSO–alginate modifications with surfactant cetylpyridinium bromide led to the formation of a polyelectrolyte complex on the interphase, allowing further enhanced the prolonged release of the drugs. The research identified that the optimal compositions for sustained drug release were organogels with compositions PVA (10%)-PVP (1%) DMSO (50%) and PVA (10%)-DMSO (50%) formulations, illustrating the transparent nature of these organogels making them suitable for ophthalmological application. Various organogels compositions (PVA-DMSO, PVA-poly(vinylpyrrolidone)-DMSO, PVA-DMSO–alginate, PVA-DMSO-PLGA, PVA-DMSO–drug–surfactant) loaded with ceftriaxone, ofloxacin, and surfactant were prepared and characterized, highlighting their potential use in antibiotic patches for wound healing. These organogels illustrate promising results for localized treatment of infections in wounds, cuts, burns, and other skin lesions.

## 1. Introduction

Hydrogels have become the most preferred material for biomedical applications (wound healing, contact lenses, and tissue engineering) today. Hydrogels are three-dimensional hydrophilic polymer networks with high water retention capacity, as well as the ability to store biological fluids [1,2].

Hydrogel matrices can be formed through both chemical and physical crosslinking, as well as a combination of these methods [3]. In the field of biomedicine, hydrogels stand out due to their ability to be compatible with living tissues and not induce toxic reactions, while also possessing strength, elasticity, stability, and biodegradability. These materials need to possess mechanical characteristics and biological functions comparable to those of the tissues they replace [4,5,6]. Additionally, hydrogels can undergo adaptive changes in their shape or stiffness in response to external stimuli, such as light, pH, temperature, electric fields, etc. [7]. Hydrogels made from natural polymers stand out for their biocompatibility, making them ideal for biomedical applications. However, they may have limited mechanical properties [8,9,10].

Furthermore, natural polymers can trigger immune and inflammatory responses. In contrast, synthetic polymer hydrogels can be designed to possess suitable mechanical properties and other characteristics required for specific applications, despite lacking inherent biological activity [11,12,13].

Recent reviews highlighted developments in organogels, which are intriguing materials made up of polymers or supramolecular networks embedded in an organic liquid phase [14]. The review emphasizes the functional versatility of organogels and their largely unexplored potential as functional materials across a broad range of applications. This potential is demonstrated by the numerous possible combinations of known organic solvents with various appropriate gelators [14].

A recent review addresses key questions regarding drug selection, loading methods, and the mechanisms of release, all of which play a pivotal role in determining the effectiveness and controlled release profiles of these systems. Case studies provide insights into how different drugs interact within organogel matrices, highlighting their potential in therapeutic applications. Organogels demonstrate significant promise in drug delivery, ranging from topical and transdermal applications to oral administration. Their adjustable properties, biocompatibility, and controlled release capabilities make them a compelling option for various therapeutic uses [15]. Organogels face limitations that hinder their pharmaceutical performance and compromise the stability of their residual architecture. Efforts are currently underway to address these drawbacks. One key issue is the purity of the component. In particular, solvent contamination can directly weaken the organogel network by disrupting essential physicochemical interactions, leading to reduced stability [16].

Synergistic interactions between different gelators in multi-component organogels are evident, as these gels demonstrate rheological and physico-mechanical parameters compared to their mono-component counterparts. This synergy allows for the optimization of the final system’s rheological properties through formulation engineering. Despite its potential, this promising concept of synergistic property enhancement has not been extensively explored in the field of biogels [17].

In the biomedical field, hydrogels (chitosan, alginate(Alg), dextrin, poly(glycolic acid), poly(lactic acid), PEG, polyvinyl alcohol (PVA), polyDMAEMA, polyHEMA, poly(lactic-co-glycolic) acid (PLGA), poly(2-hydroxyethyl methacrylate), etc.) are used for drug delivery (antibacterial, antitumor), wound dressings, contact lenses, artificial blood vessels, immunomodulation, cell encapsulation, surgical adhesives, regenerative medicine, and wound healing applications [18].

Hydrogels and organogels, in contrast to xerogels, are more flexible and resilient, allowing them to conform better to the application site. The disadvantages of xerogels in drug delivery—such as lower drug release rates, reduced swelling capacity, brittleness, limited biocompatibility, slower rehydration, and poor adhesion—make them less favorable compared to hydrogels and organogels, which offer more efficient and controlled drug delivery [19]. Thus, xerogels are often brittle and prone to cracking, which may result in mechanical failure when applied to biological tissues. This fragility can affect the reliability and consistency of drug delivery. Hydrogels and organogels, in contrast, are more flexible and resilient, allowing them to conform better to the application site.

PVA has received FDA approval as a material characterized by biocompatibility and the absence of antigenic activity. Organogels based on PVA, containing 60 wt.% dimethyl sulfoxide (DMSO), demonstrated the most promising antifreeze properties, remaining unfrozen at temperatures around −50 °C after gel formation [20,21,22].

The novelty of this work lies in the use of various polymers and combinations, which allows optimizing the properties of organogels, such as the degree of water absorption, the rate of drug release, and their resistance to degradation, which contributes to increased treatment efficacy and reduced potential side effects. The transparency of organogels may be applied for controlled drug delivery in ophthalmology, with the potential prospect of their application in other areas of biomedicine.

## 2. Result and Discussion

### 2.1. Organogel Preparation and Characterization

**Figure 1:**
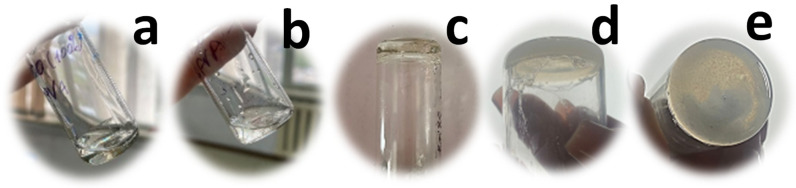
PVA solution 10 wt % in (**a**) water (sol.); (**b**) pure DMSO (sol.); (**c**) PVA 10% organogel from 60/40% DMSO/H_2_O; (**d**,**e**) PVA-PLGA10 and 4% organogel from 60/40% DMSO/H_2_O, respectively.

Solutions with 0 and 100% DMSO content remained liquid for 20 min, but were frozen during long-term storage in a freezer. The PVA-DMSO solution (0% DMSO content) showed little thawing and poor mechanical properties at room temperature, while the 100% DMSO solution retained its gel-like consistency and strength at room temperature. It follows that both water molecules and DMSO are able to interact with PVA (Figure 1). The previous report ascribes rapid gelation upon mixing of PVA solubilized in different solvents. PVA was found to be soluble in water and DMSO individually, but in water–DMSO mixtures, crystallization-induced gelation and liquid–liquid phase separation occurred. The Flory–Huggins model, incorporating three binary interaction parameters and one ternary interaction parameter, was applied to examine the cononsolvency effect, where two solvents mix to form a nonsolvent. When a composition-dependent ternary interaction parameter was included, the equilibrium crystallization line in the DMSO-rich region and the overall calculated binodal curves closely matched the experimental data [23]. The trigger force is the solvation of DMSO with water and a significant decline of free water. Thus, for 50% DMSO, the molar ratio is 1 DMSO molecule and 4.37 molecules of water, while for 60% DMSO, the molar ratio is 1 DMSO molecule and 3.6 molecules of water. Water and DMSO are thermodynamically good solvents for PVA, but DMSO–water is attributed to poor solvent.

However, in a mixture of water and DMSO, the interaction between them is stronger than with PVA, which leads to the removal of the solvation layer from the polymer chains and facilitates the formation of hydrogen bonds between the hydroxyl groups of the polymeric PVA chains. These hydrogen bonds act as physical bonds in the gel. (Figure 1) At the same time, due to the strong interaction between water molecules and DMSO, a eutectic solvent is formed, which lowers the freezing point below that of pure water and DMSO [24]. This circumstance allows gels to retain the ability not to freeze at low temperatures, while simultaneously forming gels [20,21]. The freezing point varies depending on the DMSO content. When the organogel was kept in a freezer for a long time at −20 °C, the gels (60, 70, and 80%) showed freezing, but gelation occurred, while the gel containing 60% DMSO showed transparent gelation without complete freezing. The gel containing 60% DMSO remained elastic, it could be tied and stretched without any damage. PVA containing DMSO was transparent and exhibited anti-icing properties at −40 °C, as the residual DMSO interfered with the hydrogen bonds of water, significantly slowing down or preventing freezing below the melting point [25].

The molecular weight of the polymer used to create the gels is expected to affect their properties. For example, 15% concentrated solutions of PVA with a low molecular weight (Mw < 70 kDa) form solutions that do not freeze. Therefore, for the production of durable gels, it is preferable to use PVA with a molecular weight of Mw > 80 kDa.

Thus, a solution with a higher molecular weight has a greater tendency to form a gel structure due to the chain entanglement effect. The density of this entanglement also increases with the rise in molecular weight, as a higher degree of alcoholysis leads to greater crystallinity and stronger inter- and intrachain hydrogen bonds [26]. In this work, the degree of alcoholysis for all PVA materials is 99%. The study by W. Ma et al. demonstrated that the improved crystallinity associated with increased molecular weight enhances the formation of physical hydrogels. Therefore, the differences in gel concentration in these PVA hydrogel films can be attributed to two factors: the entanglement effect and the crystallites of PVA influenced by its molecular weight [26].

The novelty of our current work consists in making the new composition of organogel PVA-PLGA-DMSO having longer kinetic of drug release due to the higher content of hydrophobic groups. Previously PLGA nanocarriers were incorporated into 5% poly(vinyl alcohol) at a crocetin:polymer ratio of 1:20 [27]. Duan et al. ascribed the formation of nanofibers poly(lactide-co-glycolide) and chitosan/poly(vinyl alcohol) utilizing simultaneous electrospun for skin reconstruction [28]. Other research by Aina et al. is devoted to the estimation of physical morphologies of PLGA scaffolds/microparticles fabricated with the help of different PVA concentrations via the water emulsion synthetic method [29]. Different formulations of PLGA microsphere/PVA hydrogel microspheres, each displaying unique drug release behaviors, were created. The PLGA microsphere/PVA hydrogel composite coatings were tested for swelling capacity, defined by the swelling ratio, and glucose permeability through the coating was examined via microdialysis [30].

PVA-PLGA-DMSO composites (hereafter referred to as PVA-PLGA) were prepared using the freezing technology, with a PLGA concentration of 4%. The addition of poly(lactic-co-glycolic acid) leads to changes in the optical properties of the gel, making it turbid. This can occur due to the formation of internal microstructures of formed interpolymer complex between PVA and PLGA, this phenomenon is facilitated due to poor solvent composition for PLGA. Intermolecular hydrogen bond formation between hydroxyl groups of PVA and carbonyl groups of glycolic and lactic acid residues takes place, which is in agreement with previous data and to some extent similar to polymeric systems. Using a dimethylformamide solution casting method, poly(DL-lactide-co-glycolic acid) was blended with poly(vinyl alcohol-co-ethylene). The appearance of a single glass transition temperature was noted, alongside a negative intermolecular interaction parameter derived from the Nishi equation and verified by the Schneider method. It was also found that increasing PLGA content in the blend reduced both crystallization enthalpy and crystallization degree, indicating the intermolecular interaction [31]. It is possible that microscopic particles or polymer aggregates are formed, resulting in light scattering and, consequently, gel turbidity.

**Figure 2:**
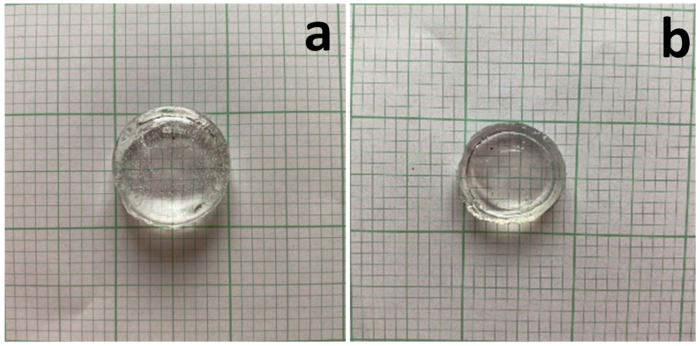
PVA 10% organogel from 60/40% DMSO/H_2_O: (**a**) diameter of freshly prepared gel d = 20 mm; (**b**) diameter of the gel after 24 h. at 25 °C diameter 15 mm, scale bar 2 mm.

Gels were obtained that remained stable when soaked in carbonate buffer (pH 7.4), while at room temperature, the PVA-PLGA-DMSO gel after 24 h storage illustrated the aging phenomenon, the moisture release, and some collapse and decrease in diameter (Figure 2). This type of organogel was subsequently used as a matrix for creating hydrogels for biomedical applications, loaded with antibiotics.

Alginate promotes the formation of a three-dimensional structure in the gel. This occurs due to alginate’s ability to form hydrogen bonds and ion-dipole interactions, enhancing the gel-forming properties of the mixture. Thus, alginate also acts as a crosslinking agent via the formation of nonstoichiometric interpolymer complex formation via hydrogen bond formation between hydroxyl groups of PVA and carbonyl groups of alginate.

**Figure 3:**
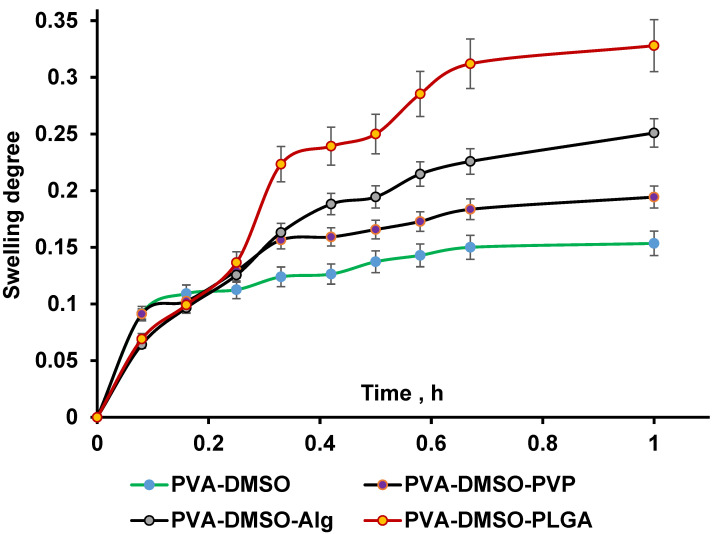
Swelling curve of PVA organogels in carbonate buffer pH 7.4.

As a result of the immersion of physically linked organogel into the aqueous solution, swelling is expected due to a significant difference in osmotic pressure. As one can observe from Figure 3, all studied organogels illustrated rapid kinetic (1 h) of swelling.

When in contact with water, polymer molecules form a hydrated network, where water fills the spaces between polymer chains. This results in an increase in gel volume because of the transition from an organogel to a hydrogel. The results indicate that water absorption characteristics of hydrogels depend on several main factors: the number of hydrophilic groups, the density of inter- and intramolecular bonds within the polymer system, and the DMSO content. The 10% organogel based on poly(vinyl alcohol) shows the lowest swelling degree in aqueous buffer at 16.6% (Table 1). Adding PVP to PVA increases the gel’s swelling degree to 19.8%, likely due to PVP enhancing the hydrophilicity of the gel matrix and its interaction with water. Incorporating Alg into PVA further raises the swelling degree to 26.56%. Alginate’s hydrophilic structure and carboxyl groups contribute to this effect, as the dissociation of carboxyl groups is significantly impacted by solvent type—being inhibited in organic solvents and enhanced at physiological pH levels above its pKa of 3.4–3.6. Consequently, the transformation from organogel to hydrogel with alginate results in more pronounced hydrophilic properties. It is likely that no interpolymer complex forms in the 10% PVA-Alg hydrogel. In hydrogels with PLGA, the swelling degree within 1 h reached 32.1% (Figure 3) and did not change after 24 h (Figure S1). Swelling data provide insights into the release rate of active pharmaceutical ingredients; as water penetrates the gel, the drug diffuses in the DMSO-buffered solution and is gradually released from the polymeric matrix. It is known that DMSO significantly improves drug penetration via the cell’s membrane. Low molecular weight compounds are also used to increase drug permeation through the skin barrier [32]. Senna et al. studied the potential of DMSO and Azone as penetration enhancers for topical delivery. In vivo, an anti-inflammatory assay illustrated that ear edema could be inhibited by drugs associated with 5.0% DMSO (53%) or 10.0% Azone (40%), leading to two times improved delivery [33].

Wang et al. investigated the penetration efficiency of a synthetic TAT peptide and the TAT fusion protein. Their study found that treatment with 10% DMSO significantly enhanced the internalization of TAT into cells, resulting in a uniform distribution throughout the cytosol and nucleus, without causing membrane disruption or noticeable cytotoxic effects [34].

In the in situ drug delivery system, the 10% PVA-DMSO organogel exhibits strong hydrophobic interactions between the PVA backbone chain and the drugs (ofloxacin or ceftriaxone), as indicated by an increase in turbidity (Table S1). A similar trend of opaque organogel formation was observed for the 10% PVA-Alg DMSO organogel with both antimicrobial agents, attributed to intermolecular complex formation that increases turbidity. Additionally, in the 10% PVA-Alg DMSO organogel, electrostatic interactions occur between oppositely charged groups and hydrogen bonds form with the polymers. According to the Cactvs 3.4.8.18 database, [35] ofloxacin has eight hydrogen bond acceptors, while ceftriaxone has fourteen, suggesting multiple cooperative binding sites with the polymer scaffold.

A similar interaction pattern occurs in the PVA-PLGA-DMSO organogel with ofloxacin and ceftriaxone, including additional hydrophobic interactions with the PVA backbone and residual non-hydrolyzed acetyl groups. In contrast, the PVA-PVP-DMSO organogel remains mostly unchanged upon drug immobilization, likely due to the dilution of PVA and the more hydrophilic nature of the gel. This could be attributed to the predominance of electron-acceptor groups in both PVP and the drugs studied. Wu and Van den Mooter examined how hydrogen bonding between the drugs (indomethacin and miconazole) and the model polymer PVPVA affects the physical stability of amorphous solid dispersions, specifically for drugs with varying tendencies to crystallize [36].

### 2.2. Drug Release Study

**Figure 4:**
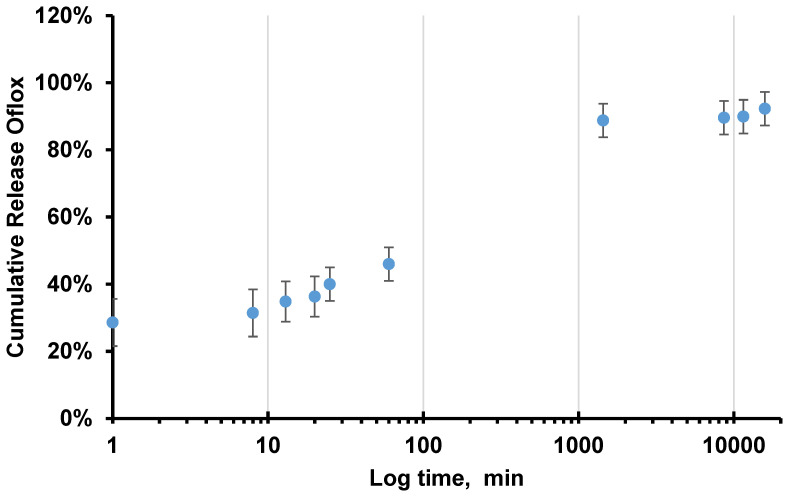
Desorption rate of ofloxacin from the PVA (10 wt.%)-DMSO gel in carbonate buffer at pH 7.4.

As was concluded from the swelling study, the PVA (10 wt.%)-DMSO organogel reaches equilibrium with buffer approximately within 1h; therefore, one can assume that the drug release and after 1 h the drug diffusion took place from PVA (10 wt.%) hydrogel, mostly based on aqueous buffer solution. In the first 25 min, approximately 45% of the drug was released, and by the next day, desorption continued, resulting in a total release of 88% of the active substance, as shown in Figure 4. In the subsequent days, there was no significant difference; thus, the majority of the substance was released within the first 24 h (Figure 4).

Our data correlate with the previous report well. The release of ofloxacin from vitamin E(VE)-modified contact lenses based on the silicone hydrogel (SHL) system (VE/SHL) was investigated. The results showed that as the concentration of ofloxacin increased from 0.1% to 0.4%, the cumulative release of the drug increased from 20% to 88%. At a concentration of 0.4%, the VE/SHL-3 contact lenses demonstrated explosive release of ofloxacin, with more than 50% of the drug released within 5 h, and cumulative release reaching 88% after 120 h [37].

The explosive release of ofloxacin was also observed. This suggests a similar release mechanism as seen with the lenses, where the rapid release of the drug was attributed to physical diffusion resulting from the concentration gradient both inside and outside the lenses [37,38].

This can be explained by the fact that once the dissolution medium penetrates the polymer matrix, it triggers swelling of the system due to osmotic pressure difference as was aforementioned in detail. This leads to the diffusion of the drug molecules into the external environment. A biphasic release profile of ofloxacin was observed. The initial phase involved a rapid release of the drug (burst effect) [23], with 40–50% of the drug released within 60 min, followed by a phase of slow and sustained release from the PVA hydrogel matrix (Figure S1). The slow-release phase gradually extended up to 24 h. In the hydrogel systems, 10% PVA-PLGA, 10% PVA-Alg, and 10% PVA hydrogen binding with both drugs were stronger due to a higher degree of ionization of drugs in water compared to the DMSO–water solution, which resulted in enhanced electrostatic interactions between oppositely charged groups.

Previously, it was reported the cumulative release of ofloxacin from gellan–ofloxacin films of 96 ± 2% in phosphate buffer and 36 ± 2% in distilled water [39].

The initial sharp release of ofloxacin from the matrix is attributed to rapid swelling, as was discussed before. The swelling brought about larger pores higher the diffusion of drug molecules that are incorporated in the matrix. From a practical point of view, the observed initial burst drug release is often desirable for quickly achieving optimal therapeutic concentrations. The subsequent phase of slow release is primarily due to the diffusion of drug molecules trapped in the central part of the matrix. This process helps maintain the drug concentration in the desired range for an extended period, enhancing the drug’s effectiveness at the site of action.

To achieve a more prolonged desorption of ofloxacin, further modification of the hydrogel matrix is necessary.

**Figure 5:**
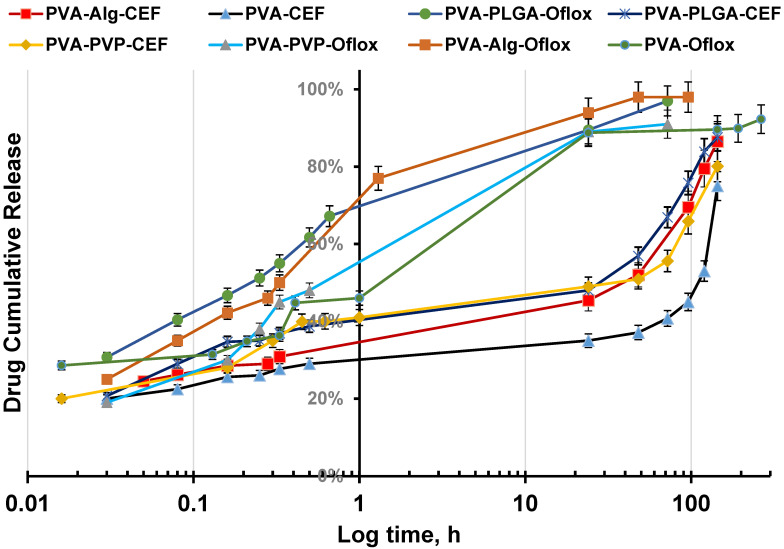
Kinetic of drug release from various PVA organogels in carbonate buffer at pH 7.4 relatively initially loaded concentration.

Numerous factors play a role in the mechanism of drug release from PVA-based gels. This swelling creates larger voids and pathways for the diffusion of drug molecules from the gel matrix. The release of ceftriaxone occurred slower compared to ofloxacin; in the first 30 min, approximately 30% of the drug was released, and around 50% of the substance was released within the first 24 h (Figure 5). The decrease in DMSO content of the gel led to a more enhanced possibility of hydrogen bond formation between ceftriaxone PVA, Alg, and PLGA. Comparison of the swelling degree with the kinetic of ceftriaxone release does not correlate, and one can conclude that the porosity insignificantly affects the diffusion. Rapid drug release from the gel at the initial stage is related to excellent drug solubility in DMSO–water mixture. This indicates a more prolonged action for ceftriaxone, which continued for up to 6 days (Figure 5).

Concentration gradients drive the diffusion of ceftriaxone molecules through the swollen hydrogel matrix. The size and charge of the drug molecules, as well as the porosity and size of the hydrogel matrix cells, influence the rate of this diffusion. The polymer matrix of the hydrogel is subject to degradation due to hydrolysis, which can compromise its integrity and lead to the release of encapsulated drug molecules [15,38]. It is worth noting that the experimental setup should checked from various angles via a significant number of control experiments to create an appropriate design. Previously, it was shown that the release of the antibiotic ceftriaxone from the encapsulated hydrogel GA-TG-cl-polyAAm occurred faster in a buffer with pH 7.4 compared to a buffer with an average pH of 2.2, due to the domination of hydrogen bonding with the substance or due to significantly higher kinetic of the drug hydrolysis at pH 2.2. It was found that the diffusion of the drug was non-Fickian, as indicated by the diffusion coefficient values: 0.53 in the pH 2.2 buffer and 0.65 in the pH 7.4 buffer. Under non-Fickian conditions, the relaxation rate of the polymer chains was comparable to the drug diffusion rate. Additionally, it was observed that in the early stages, the diffusion of ceftriaxone from the hydrogel occurred faster than in the later stages [39].

Poly-3-hydroxybutyrate (P3HB) and poly-3-hydroxybutyrate/polyethylene glycol (P3HB-PEG)-based microparticles with a size 3.84 to 6.51 µm loaded with ceftriaxone illustrated also quasi-Fickian diffusion [40].

It is important to note that the molecular weight and lipophilicity of ceftriaxone log P −0.25 [41] slightly exceed those of ofloxacin Log P −0.39 [42]. However, the molecular weight differ significantly 1323.2 g/mol vs. 361.4 g/mol as do the hydrogen bond donor and hydrogen bond acceptor groups 11 & 35 and 1 & 8 bonds, respectively. Ceftriaxone is an antibiotic from the cephalosporin group, which has a larger molecular structure compared to ofloxacin, a fluoroquinolone. The greater molecular weight of ceftriaxone may hinder its diffusion through the polymer matrix of the hydrogel, resulting in a slower release.

**Figure 6:**
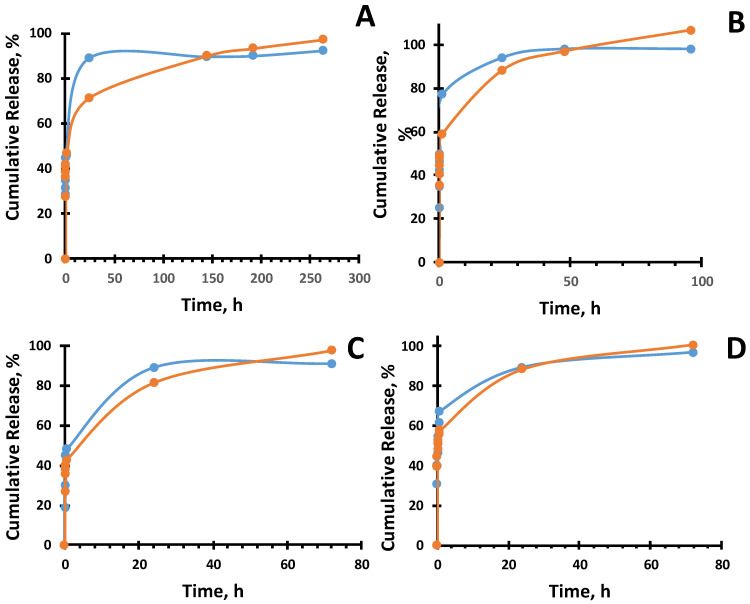
Korsmeyer–Peppas models for the ofloxacin cumulative release from organogel, orange line theoretical model; blue line experimental data: (**a**) 10% PVA-DMSO; (**b**) 10% PVA-DMSO-Alg (**c**) 10% PVA-DMSO-PVP; (**d**) 10% PVA-DMSO-PLGA.

**Figure 7:**
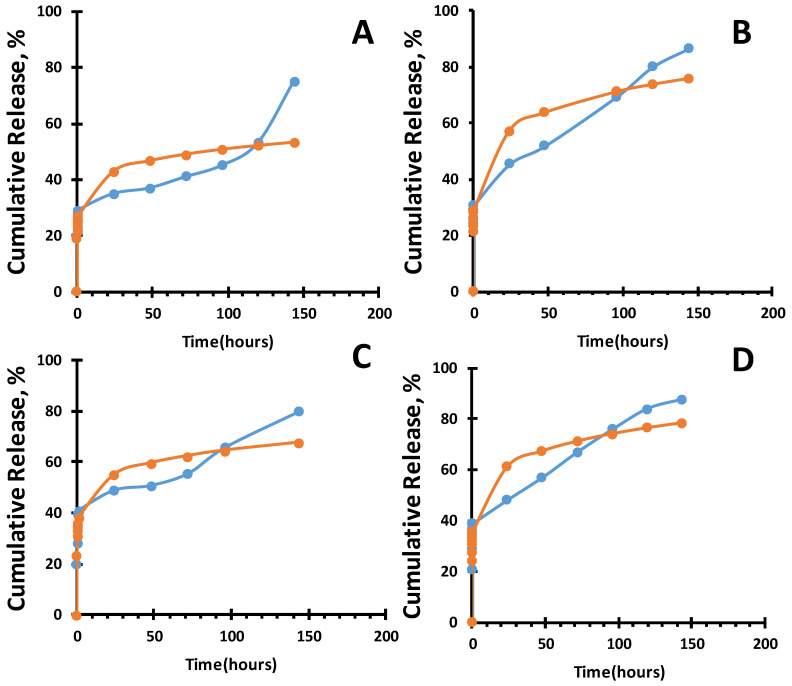
Korsmeyer–Peppas models of ceftriaxone cumulative release from organogels orange line theoretical model; blue line experimental data.: (**a**) PVA-DMSO; (**b**) PVA-DMSO-Alg; (**c**) PVA-DMSO-PVP (**d**) PVA-DMSO-PLGA.

Several mathematical models exist to describe the diffusion of the drug: first-order release kinetics, the Higuchi model, the Hopfenberg model, the Ritger–Peppas model, the Korsmeyer–Peppas model, and others [43].For all datasets, a high correlation coefficient (R^2^) was observed in the range of 0.95 to 0.98, indicating a strong relationship between the experimental data and the applied equation (Figure 6 and Figure 7). The study showed that the release profile of ofloxacin and ceftriaxone from various types of PVA closely follows the Korsmeyer–Peppas model, with the release exponent n (diffusion coefficient) ranging from 0.11 to 0.16 (Table 2). This value suggests a diffusion release process consistent with quasi-Fickian diffusion (Table 3). Quasi-Fickian diffusion refers to a type of diffusion characterized by a rapid initial diffusion process that subsequently slows down. In the context of drug diffusion from the hydrogel polymer matrix, this means that initially, the drug is released from the hydrogel matrix relatively quickly, but over time, the process slows, and the release rate decreases [44].

Fickian diffusion of the drug typically occurs from swollen polymer matrices. The release profile and the chosen kinetic model indicate that at the initial stage of release, the matrix swells, and the majority of the release occurs via drug diffusion and polymer matrix dissolution. Unlike full-Fickian diffusion, where the substance is entirely retained in the matrix or medium, quasi-Fickian diffusion means that part of the substance can still diffuse freely while another part remains fixed within the matrix or medium [44].

The high values of the correlation coefficient (R^2^ from 0.95 to 0.98) for the drug release model suggest the significance of this model for the release process of ofloxacin and ceftriaxone. In the case of Fickian diffusion, the change in concentration of the substance is directly proportional to the concentration gradient at various points in the medium. However, non-Fickian diffusion describes processes that do not conform to this law, which may be caused by various factors, such as changes in matrix properties or interactions between the diffusing substance and the matrix [44].

An analysis of the obtained data compared with literature values for the diffusion coefficient revealed that in most sources, n was equal to 0.54–0.6, indicating Fickian diffusion [45]. Alginate-containing gels showed a coefficient of 0.1–0.2 [46], which is similar to experimental data. This suggests that differences in coefficients across studies may arise even if the polymer matrix is the same (e.g., PVA-containing hydrogel); different studies may utilize varying proportions or types of excipients that can influence the properties of the hydrogel, including its structure. Various experimental conditions, such as drug concentration and particle size, may also impact the diffusion rate and, consequently, the diffusion coefficient.

The release profile of the substance from gellan–ofloxacin films cross-linked with CaCl_2_ with weight 10 mg, 4.0 ± 0.2 mm in diameter, and 0.5 ± 0.1 mm thickness corresponded to non-Fickian diffusion (n = 0.59) in distilled water and to Fickian transport of type II (n = 0.95) in phosphate buffer [47], whose data are more practical for biomedical application.

### 2.3. Kinetic of Release Antibiotics from PVA-DMSO–Surfactant Complex

Other substances present in the solution can penetrate the micelles, thereby increasing the overall solubility of the substance in water through a process called micellar solubilization. Micelles consist of a hydrophobic core that serves as a reservoir for hydrophobic drugs and a hydrophilic shell that influences their pharmacokinetic behavior. The micellization of cetylpyridinium bromide and levofloxacin hemihydrate in solutions of additive mixtures (NaCl, NaOAc, NaBenz, 4-ABA, and urea) was studied. The Δ*H*0m and Δ*S*0m data generated for the association of the CPBr and levofloxacin mixture illustrated the characteristic interaction forces due to probable ion–dipole, dipole–dipole, and hydrophobic between CPBr and the drug [48].

Previously, PVA–cetyltrimethylammonium bromide (CTAB) interaction was monitored at 272 nm using UV spectroscopy. The absorbance of PVA-CTAB increased at a lower concentration due to the solubility of the polymer and it was confirmed that the ammonium salts’ distinctive groups were integrated into the PVA [49].

Very recently, PVA-PBr complex formation was studied in water and aqueous solution of various alcohols (EtOH, 1-PrOH, and 2-BuOH) via the conductivity measurement. The CMC values of PVA and CPBr were increased via an increase in alcohol concentration. The aggregation of the CPBr-PVA system was hindered in aqueous–alcoholic medium compared to that in water [50]. PVA hydrogels containing cetylpyridinium chloride were synthesized by irradiation. If a positively charged organic antibacterial agent is loaded into a gel with polar groups, suppressed release is observed due to electrostatic repulsion [51].

Our approach was to create a drug delivery system with prolonged release. Taking into account previous research in the field of hydrogel–surfactant complexes, novel systems PVA-DMSO-Alg-CPBr-Oflox and PVA-DMSO-Alg-CPBr-CEF were evaluated.

PVA-DMSO-Alg-Oflox and PVA-DMSO-Alg-CEF gels were immersed in the CPBr aqueous solution resulting in polyelectrolyte complex formation between opposite charged carboxyl group of Alg and positively charged nitrogen of CPBr. Previously, CPCl was applied as a cross-linker for Alg for stable polyelectrolyte–surfactant complex nanoparticles preparation. The substance release from Alg-CPCl ibuprofen–nanoparticles was not hindered by Alg, and had comparable release kinetics from ibuprofen–CPCl solubilities [52].

A generated polyelectrolyte shell prevents regular drug diffusion due to the association of hydrophobic tails with a backbone of PVA.

Hydrophobic drugs can be physically encapsulated in the core of the micelles and transported at concentrations significantly exceeding their own solubility in water. The hydrophilic blocks can form hydrogen bonds with the aqueous environment, creating a dense shell around the micelle core.

**Figure 8:**
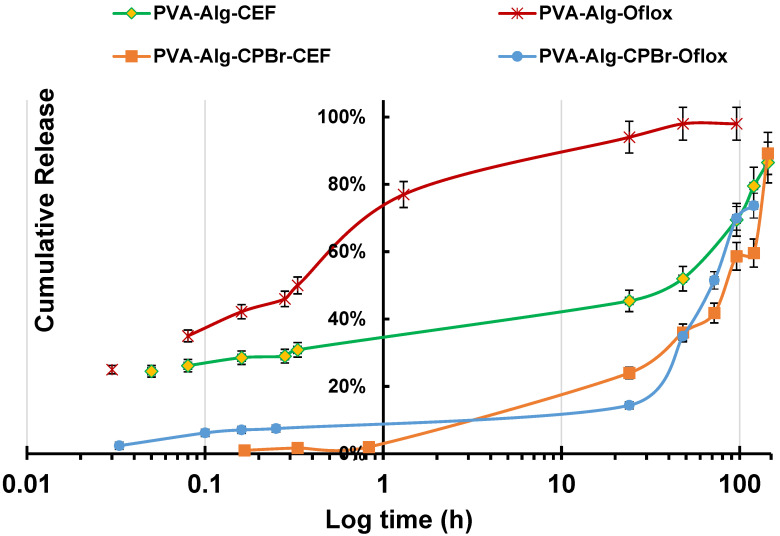
Kinetic release of Oflox and ceftriaxone from PVA-DMSO-Alg-CPBr-Oflox and PVA-DMSO-Alg-CPBr-CEF, PVA-DMSO-Alg-Oflox, PVA-DMSO-Alg-CEF.

During the swelling of the PVA-DMSO-Alg-drug, an additional complex of the antibiotic with surfactant micelles was formed, indicating the encapsulation of substances in the micelle core and thus increasing the drug release time. In the first 24 h, 14.4% of ofloxacin was released from PVA-DMSO-Alg-CPBr-Oflox, while PVA-DMSO-Alg-Oflox 94% of ofloxacin was released in the same period (Figure 8).

Within 15 min, 29% of ceftriaxone of was diffused out from the PVA-DMSO-Alg-CEF and detected using spectrophotometry, whereas only 2% was released from the PVA-DMSO-Alg-CPBr-CEF complex. This suggests that by incorporating substances into surfactants, prolonged drug release from the polymer matrix can be achieved.

The impact of cetalkonium chloride in oil-in-water microemulsions encapsulated in pHEMA-based contact lenses was examined to facilitate the controlled delivery of sodium diclofenac. The control contact lenses released 80% of the loaded sodium diclofenac within 12 h, whereas the release of diclofenac decreased to approximately 70% over the same period when surfactant polyoxyethylene-10-oleyl-ether Brij 97 was added [53].

A hydrogel was prepared through UV-crosslinking of methacrylated amphiphilic inulin derivatives and vitamin E. The methacrylated micelles were subjected to UV irradiation to create what they referred to as “nanosieves”. The vitamin E release rate was found to be nearly constant throughout the experiment, indicating controlled release due to both the diffusion of drug molecules through the hydrophilic layer and hydrophobic interactions within the hydrophobic core [54].

### 2.4. Antibacterial Activity of Organogels

**Figure 9:**
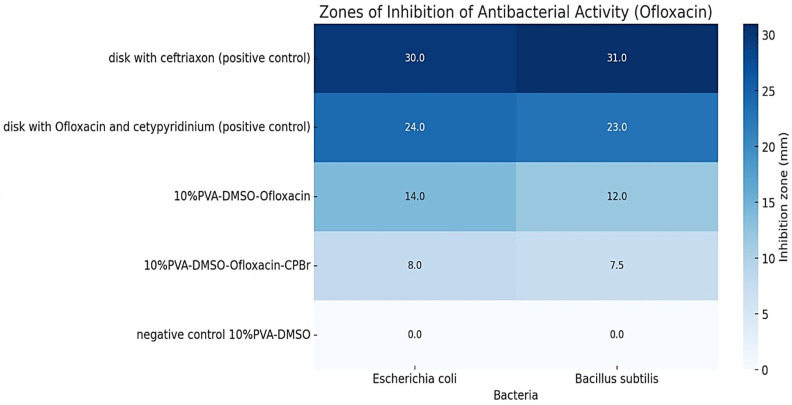
Zones of inhibition of antibacterial activity (ofloxacin).

Antimicrobial analysis showed that drug-loaded organogels released the in situ incorporated ofloxacin, which was active against *Escherichia coli*, *Bacillus subtilis*, and *Staphylococcus aureus* (Figure 9). As expected, polyelectrolyte complex PVA-DMSO-Alg-CPBr-Oflox revealed almost two times less inhibition zone due to significantly slower diffusion compared to PVA-DMSO-Alg-Oflox (Figure 8).

**Figure 10:**
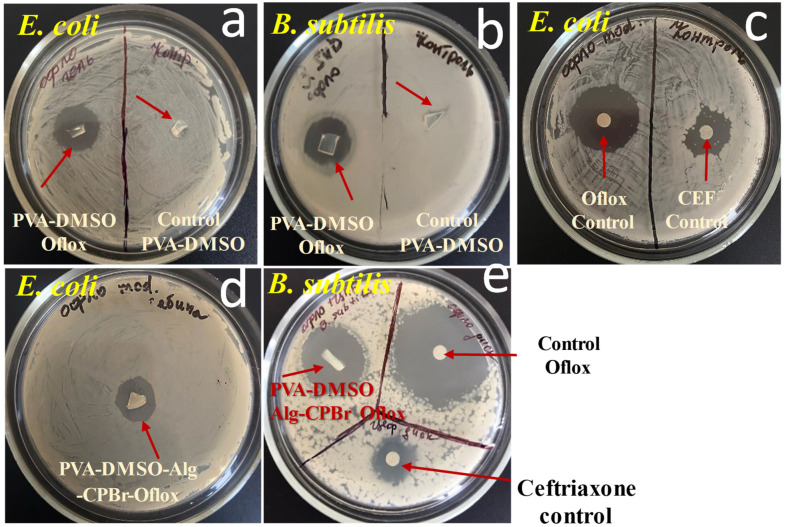
Antibacterial activity of organogels via method diffusion to agar gel: (**a**) 10%PVA-DMSO-Oflox; (**b**) 10%PVA-DMSO-Oflox; (**c**) control samples paper disk with ofloxacin and cetylpyridinium bromide; (**d**) 10%PVA-DMSO-AlgoOflox-CPBr; (**e**) 10%PVA-DMSO-Alg-Oflox-CPBr, (control sample) disk with Ofloxacin, (control sample) disk with Ceftriaxone against *B. subtilis*.

**Figure 11:**
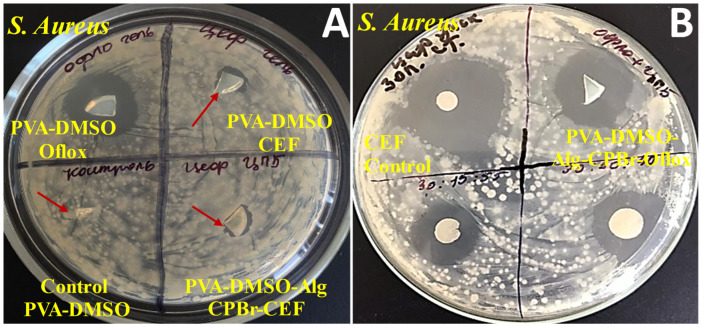
Antibacterial activity of drug delivery system based on organogel against *S. Aureus*: (**A**) 10%PVA-DMSO-Oflox, 10%PVA-DMSO-Ceftriaxone, 10%PVA-DMSO-Alg-CPBr-Ceftriaxone; and (**B**) control samples paper disk with ceftriaxone, 10%PVA-DMSO-Alg-Oflox-CPBr.

**Figure 12:**
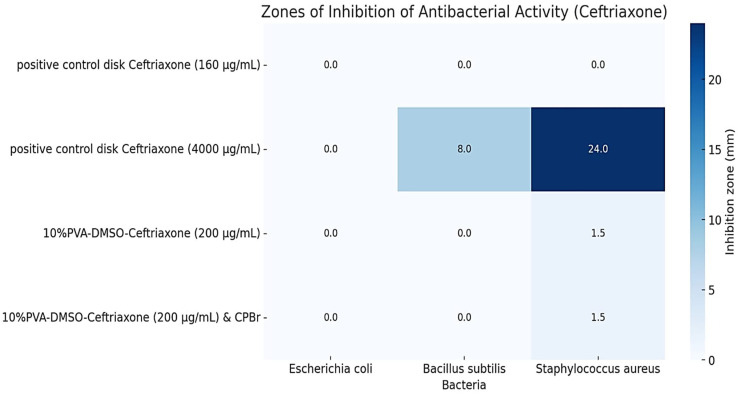
Zones of inhibition of antibacterial activity (ceftriaxone).

The organogels containing ofloxacin exhibited a larger zone of inhibition against *E. coli* compared to *B. subtilis*, indicating that *E. coli* is more sensitive to the antibiotic ofloxacin, with a slight difference (Figure 10 and Figure 11). The results of the study demonstrate that the presence of cetylpyridinium bromide in solution to some extent improves the antibacterial activity of the gel PVA-DMSO-Alg-CPBr-CEF, (Figure 12) as the kinetic release is significantly slower (Figure 8). This illustrates the stability of the polyelectrolyte complex PVA-Alg-BPBr in physiological pH. The advantage of incorporating it into the polymer matrix PVA-DMSO-Alg-BPBr is that it allows for the controlled release of the substance, which can enhance its effectiveness and duration of action. PVA hydrogels containing cetylpyridinium chloride revealed antibacterial activity against *Escherichia coli*. The capacity adsorption of the surfactant was estimated as a 4.1×0^−2^ CPC/PVA-OH molar ratio [51].

Carbopol 940 loaded with ofloxacin in in-vitro diffusion and ex-vivo diffusion through goat skin studies illustrated an 88 ± 1.8% and 76 ± 2.5% drug release, respectively. The developed formulation was effective with an inhibition zone for *E. coli* and *St. aureus* of 27 and 33 mm, respectively [55]. Polyelectrolyte–surfactant complexes (PESCs) based on pre-hydrolyzed polyacrylonitrile and quaternary ammonium salts membranes revealed antibacterial activities against both Gram-negative *E. coli* and Gram-positive *St. aureus* [56]. Alginate nanoparticles loaded with an amphiphilic cationic drug cetylpyridinium chloride and cross-linked with zinc cations have two acting antimicrobial agents with potential strong activity [57].

Previously, the possibility of the synergetic effect of the combination of silver nanoparticles with surfactant was illustrated. The bactericidal effect against the strain of *Staphylococcus aureus* was observed at a concentration of 0.005.9 mM solution of benzalkonium chloride (0.2%) and 0.97 mM AgNPs [58].

## 3. Conclusions

For the first time, we report the possibility of interpolymer complex formation PVA-PLGA, PVA-PVP, and PVA-Alg for organogels, as well as hydrogel preparation without the use of covalent cross-linking. The obtained polymeric gels have perspective application in terms of drug delivery systems with enhanced cell membrane permeability due to DMSO presence. The studies found that the optimal composition of hydrogels for prolonged release is a formulation of ofloxacin or ceftriaxone in situ loaded into (10%) PVA-Alg-CPBr, and following encapsulation into polyelectrolyte complex between Alg and CPBr surfactant on the surface. Various organogels were developed during the research (PVA-DMSO, PVA-PVP-DMSO, PVA-Alg-DMSO, PVA-PLGA-DMSO) containing both ceftriaxone and ofloxacin, which are widely used antibacterial drugs. The addition of a polyelectrolyte complex with surfactant achieved a prolonged release effect, indicating the potential use of the material as a basis for antibiotic patches intended for healing effects. The hydrogel with antibiotics can be used for local treatment of wounds, cuts, burns, and other skin injuries where an infectious process is present, as well as for applications in ophthalmology.

## 4. Materials and Methods

### 4.1. Materials

Poly(vinyl alcohol) (PVA) (Mw146 to 186 kDa) and PVA (Mw 30–70 kDa), alginate 99%, poly(vinylpyrrolidone) (40 kDa), cetylpyridinium bromide (CPBr) 99.5% (C21H38BrNH2O) were obtained SigmaAldrich (Saint Louis, MO, USA) lactic acid 85% in water solution, creatinine 99%, glycolic acid 99.5% was purchased from (Sigma–Aldrich Ltd., Dorset, UK)). Ofloxacin (100 mL infusion solution 2 mg/mL, active ingredient: ofloxacin, excipients: sodium chloride, water for injections (endotoxin and pyrogen-free)), chloroform 99.8% dimethyl sulfoxide DMSO 99%, ethylacetate 99.5%, Ceftriaxone-AKOS (sodium salt hydrate) sterile ampule (endotoxin and pyrogen-free) were obtained from JSC Synthez (Kurgan, Russia). Na_2_CO_3_ × 10H_2_O 99.5%, ethanol 90%, and nutrient agar (TM Media) were obtained from local JSC LabrPharma (Kazakhstan, Almaty).

### 4.2. Synthesis of PLGA

#### 4.2.1. Synthesis of Oligolactic-Glycolic Acid

For the synthesis of medical-grade biodegradable poly(lactic-co-glycolic acid), lactic acid and glycolic acid undergo co-polycondensation catalyzed by biomass-derived creatinine using procedure ascribed in patent US US20140142275A1 from 2011 [59].

A commercially available creatinine was utilized as the catalyst. Industrial-grade lactic acid (85% aqueous solution) and glycolic acid (95%) serve as monomers in a molar ratio of 1 to 1 was used. The process involves a two-step, solvent-free polycondensation, resulting in poly(lactic-co-glycolic acid) with high biosafety for medical applications.

A reactor was loaded with 45 g of an 85% aqueous solution of industrial-grade lactic acid (LA) and 38 g of glycolic acid (GA) with a 95% purity. The system was then subjected to three cycles of vacuumization and argon purging. Under normal pressure in an argon atmosphere, the mixture was heated to 130 °C and dehydrated for 3 h. Subsequently, the pressure in the reactor was reduced to 100 Torr, and the reaction continued at 130 °C for another 3 h. Finally, the pressure was further lowered to 30 Torr, with the reaction proceeding at 130 °C for an additional 3 h.

#### 4.2.2. Synthesis of Polylactic-Glycolic Acid

An amount of 60 g of oligolactic-glycolic acid and 0.6 g of creatinine catalyst were introduced into the reactor. The pressure was reduced to 10 Torr, and the mixture was heated to 160 °C for 170 h [60]. After completing the reaction, the reactor was allowed to cool to room temperature. The solid polymer was dissolved in chloroform and then the polymer was precipitated in an ethanol solution, the precipitated PLGA was then dissolved in chloroform and precipitated in ethanol in order to remove oligomers and impurities.

### 4.3. Synthesis of PVA-DMSO Ogranogels

(a) The preparation of organogels was carried out according to the previously ascribed method [22]. To form PVA organogel, two 10 wt.% solutions of PVA (Mw146 to 186 kDa) were dissolved in pure DMSO and distilled water were mixed under vigorous stirring and heating (95 °C); afterward, solutions were cooled down to room temperature. These PVA solutions were mixed in different ratios to obtain 60%, 70%, and 80%, 0% and 100% of PVA in DMSO, and the solution was placed in the freezer at −20 °C. Transparent organogels formed within just 3 min at −20 °C, which could be visually observed by the gel remaining at the bottom of the bottle after it was turned upside down (Figure 1).
(b) To prepare organogels using PVA (Mw 30–70 kDa), DMSO and aqueous solutions with 15 wt.% dissolved PVA were made, referred to as PVA-DMSO and PVA–water solutions, following a similar method as previously used. These solutions were mixed in different proportions and stored in a freezer at −20 °C, with DMSO concentrations of 60%, 70%, and 80%. Gelation did not occur after 10 min. Nor after 3 days of freezing at −20 °C. The same experiment was repeated by mixing 10 wt.% PVA (Mw 30–70 kDa) in water and 10 wt.% PVA (Mw 30–70 kDa) in DMSO 60%, 70%, and 80%, respectively, which did not result in organogel formation.
(c) Introducing a third component, such as alginate 1 and 2 wt.% or poly(lactic-co-glycolic acid) at concentration of 1, 2, 4 wt.% did not produce stable organogel using 10 and 15% PVA (Mw 30–70 kDa) in 50% DMSO solution. It is likely that polyvinyl alcohol with a molecular weight of Mw 30–70 kDa cannot form a robust three-dimensional network, unlike higher molecular weight PVA (Mw 146 to 186 kDa), or that its interaction with DMSO and water hinders organogel formation.
(d) In the preparation of 10 wt.% PVA-Alg (1%)-DMSO (50%) organogel, dry alginate was added to the initial 10 wt.% PVA–water solution at a concentration of 1%. This resulted in a solution containing alginate 10 wt.% PVA and Alg 2 wt.% was mixed 1 to 1 with 10 wt.% PVA-DMSO (100%) that subsequently began to gel with the PVA-DMSO solution at room temperature (25 °C). The transparent gels formed within just 3 min at a temperature of -20 °C, which could be visually observed by the gel remaining at the bottom of the bottle after it was flipped.
(e) Aqueous solutions of 10 wt.% and 2% PVA-polyvinylpyrrolidone (PVP) were prepared by dissolving PVP in PVA–water at 90 °C with slow stirring for 2 h. The solution was then allowed to swell and cool at room temperature (25 °C) for 4 h.

For the preparation of (10 wt.% ) PVA-PVP(1 wt.% ) -DMSO gels (hereafter PVA-PVP), 10 wt.% PVA containing 60% DMSO was added to the obtained aqueous solution, resulting in a transparent gel forming in the freezer within 5 min. The PVP content in this mixture was 1%.

### 4.4. Swelling of Gels

The swelling properties of ofloxacin and ceftriaxone-free PVA-DMSO, PVA-PVP, PVA-Alg, and PVA-PLGA samples in carbonate buffer (pH 7.3) were observed to measure the swelling percentage, which plays a major role in achieving the drug release and entrapment characteristics of the hydrogel. These hydrogels were loaded into mesh syringes and dropped into 50 mL of carbonate buffer. After every 5 min, the meshes were removed, and blotted with filter paper and the change in weight was determined. This process was repeated until a constant weight was achieved [61]. The swelling ratio was measured using the corresponding Equation (1) below.

(1) $$W=\frac{\left(Ws-Wd\right)}{Wd}*100\%,$$

where W_s_ is the mass of the swollen gel;

W_d_ is the mass of the dry gel.

A graph was constructed showing the dependence of time on the swelling coefficient of the gel in a certain period of time.

### 4.5. In Situ Drug Loading in Organogel

Pharmaceutical substances were incorporated into the gels through physical adsorption during the preparation stage in the aqueous PVA, PVA-Alg, and PVA-PVP solutions. A stock solution of ceftriaxone, dissolved in 0.9% sterile NaCl, was added to the individual aqueous 15% PVA solution with a ratio of 1 to 3 and then adjusted by water to obtain a final aqueous solution of 10% PVA with drugs; afterward, 10% PVA in pure DMSO were added to separate vials, and the gels filled with drugs were left in the freezer for 1 h. The concentrations of the drugs are presented in Table 4. During the freezing process, the organogel remained transparent and did not freeze due to freezing depression and the freezing point DMSO-H_2_O 50–50% being below −50 °C. The final composition of the gel contains 50% DMSO and 50% water; taking into account that PVA creates a hydrating shell around hydrophilic functional groups of polymers; the content of free water is lower compared to DMSO; therefore, we call it organogel.

An amount of 200 μL ofloxacin was diluted to 5 mL with solutions 0.9% sterile NaCl (dilution factor 25 times). This solution was diluted with 15% aqueous PVA solution with a ratio of 1 to 3 and then mixed with 10% PVA in pure DMSO with the following storage in the freezer at approximately −20 °C.

### 4.6. Gels Modification with Surfactant Shell

Cetipyridinium bromide (C_21_H_38_BrN), the surfactant solution, was prepared to take into account the critical micelle concentration of 7.5 × 10^−3^ mol/L. For that, 0.15 g of dry powder was dissolved in 50 mL of distilled water and heated until completely dissolved, and the obtained pH solution was equal to 6.9. One simple and effective method involves the physical modification of antibiotics, for example, by immobilizing them in micelles of organic surfactants. These surfactants themselves possess bacteriostatic and bactericidal properties [59]. PVA (10 wt.%) -DMSO-Alg (1 wt.%), PVA (10 wt.%) -DMSO-Alg (1 wt.%)–ofloxacin, and (10 wt.%) PVA-DMSO (50%)-Alg (1 wt.%)–ceftriaxone were immersed in a solution of cetylpyridinium bromide. The swelling degree monitoring of these gels was carried out for 1 h, until it reached the swelling equilibrium.

Subsequently, a kinetic release study of ofloxacin and ceftriaxone from PVA(10 wt.%) -DMSO-Alg (1 wt.%)–ofloxacin and PVA(10 wt.%) -DMSO (50%)-Alg (1 wt.%)–ceftriaxone in carbonate buffer (pH 7.4) was conducted, using a spectrophotometer.

### 4.7. Kinetic of Drug Release Study

pH meter BIOBASE pH-210 with corresponding calibration solutions 4.01, 7.01, and 9.01 was used prior to pH measurement and for buffer preparation.

UV spectra of drugs and kinetic of release were investigated using SHIMADZU UV-2600i, and SHIMADZU 1900i using corresponding buffer solution for a baseline recording using a glass cuvette. UV spectra and calibration curves for ofloxacin and ceftriaxone are presented in Figures S2 and S3. Release studies were conducted by immersing the drug-loaded gels in a carbonate buffer (pH 7.4). The dynamic concentration of the drug in the carbonate buffer (release medium) was analyzed by measuring the optical density of the release medium at a wavelength of 293 nm for ofloxacin and 290 nm for ceftriaxone every 5 min, and then every specified hour/day until consistent absorption was achieved after three consecutive measurements. A control study was conducted by immersing a lens without the drug in the carbonate buffer, and the optical density was measured at the same wavelengths using the UV–Vis spectrophotometer. The drug release experiment was conducted over a period of one to five days.

To study the release kinetics of ofloxacin and ceftriaxone from polymer matrices, the Korsmeyer–Peppas model was employed:

(2) $$\frac{Mt}{Ma}\;=\;K\;\times \;t^{n}$$

where M_t_/M_a_ is the fraction of the drug released at time t, K is the release rate constant possessing units of t^n^ incorporating structural and geometric characteristics of the delivery system, and n is the release exponent indicative of the mechanism of transport of substance through the polymeric matrix. The value of n is used to characterize the mechanism of drug release [43]. The calculated data using the Korsmeyer–Peppas model were obtained using Microsoft Excel Office software 2016 mso (16.0.4549.1000).

### 4.8. Antibacterial Activity via Agar Diffusion

The minimum inhibitory ofloxacin concentration against microorganisms according to literature data is 2 µg/mL [62], while for ceftriaxone it ranges from 0.03 to 2 µg/mL [63].

Antimicrobial activity was estimated according to the standard method. *Escherichia coli* and *Bacillus subtilis* were cultured and inoculated on nutrient agar. Gels containing ofloxacin and ceftriaxone were placed on agar inoculated with *E. coli* and *B. subtilis*. A PVA-DMSO without any drug was also placed on the agar seeded with bacteria to serve as a negative control [64]. The antimicrobial activity of gels, PVA-DMSO-Alg-CPBr–ofloxacin as well as PVA-DMSO-Alg-CPBr–ceftriaxone, was assessed. For positive control, disks containing ofloxacin (40 µg/mL) and ceftriaxone (160 µg/mL) were used. All procedures were conducted in a laminar flow hood to prevent contamination. The agar gel Petri dishes with tested drug-containing PVA-DMSO and PVA-DMSO-Alg-CPBr gels were incubated at 37 °C for 24 h. After incubation, Petri dishes were inverted and placed in a well-lit area against a black background, and the diameter of the inhibition zone was measured in triplicate.

### 4.9. Statistical Analysis

Microsoft excel 2016 mso (16.0.4549.1000) was used to evaluate the data. The mean values ± standard deviation was used to present quantitative data.

## Figures and Tables

**Table 1:** Swelling ratio of organogels with different compositions after 24 h incubation at standard conditions.

Type of Gel	Mean Swelling Degree, %	Standard Deviation, %
10% PVA-DMSO	16.6	±0.4
10% PVA-DMSO PVP (1%)	19.8	±0.8
10% PVA-DMSO-Alg (1%)	26.56%	±1.4
10% PVA-DMSO-PLGA (4%)	32.1%	±0.7

**Table 2:** Kinetic parameters of ceftriaxone and ofloxacin release from different types of gels obtained using the Korsmeyer–Peppas model, where n is the release exponent indicative of the mechanism of transport of substance through the polymeric matrix.

Type of Gel	n	k, %/min ^n^	R^2^
PVA-DMSO-Oflox	0.130	47.2	0.97
PVA-Alg-DMSO-Oflox	0.137	57.1	0.97
PVA-PVP-DMSO-Oflox	0.165	48.0	0.98
PVA-PLGA-DMSO-Oflox	0.119	60.6	0.98
PVA-DMSO-CEF	0.120	29.2	0.95
PVA-Alg-DMSO-CEF	0.158	34.5	0.97
PVA-PVP-DMSO-CEF	0.115	38.0	0.96
PVA-PLGA-DMSO-CEF	0.139	39.3	0.97
PVA amoxicillin and hydrochloride tetracycline [45],	0.54–0.6	-	0.95–0.97
hydrogels PVA-Alg (2.5%) [37]	0.10–0.20	20–29	-

**Table 3:** Characterization of Drug Transport Mechanisms [26].

Release Exponent (n)	Mechanism of Drug Transport	Rate as a Function of Time
n < 0.5	Quasi-Fickian Diffusion	t^n^
0.5	Fickian Diffusion	t^0.5^
0.5 < n < 1.0	Anomalous (Non-Fickian) Diffusion	t^n−1^
1.0	Case II Transport	time-independent
>1	Super Case II Transport	t^n−1^

**Table 4:** Content of active pharmaceutical ingredient in various PVA–polymer organogels in 50% DMSO.

№	Composition of the Organogels	API	API Concentration µg/mL
1	10 wt.% PVA-DMSO	Ofloxacin	40
2		Ceftriaxone	7
3	10 wt.% PVA DMSO-Alg 1 wt.%	Ofloxacin	8.6
4		Ceftriaxone	10.013
5	10 wt.% PVA-DMSO PVP 1%	Ofloxacin	16
6		Ceftriaxone	9.785
7	10 wt.% PVA-DMSO-PLGA 4%	Ofloxacin	11
8		Ceftriaxone	13

## Data Availability

The original contributions presented in this study are included in the article/supplementary material. Further inquiries can be directed to the corresponding author.

## Supplementary Materials

The following supporting information can be downloaded at: https://www.mdpi.com/article/10.3390/gels10110753/s1, Figure S1: Swelling curve of PVA organogels in carbonate buffer pH 7.4; Figure S2: UV spectra of aqueous solution of (a) ofloxacin; (b) ceftriaxone; Figure S3: Calibration curve for ceftriaxone at 290 nm and ofloxacin at 293 nm (SHIMADZU 6000i); Table S1: A relative visual transparency level of organogels 10% PVA without and with drugs ofloxacin and ceftriaxone.
