# Supplementary material for: Poly(Vinyl Alcohol) Drug and PVA–Drug–Surfactant Complex Organogel with Dimethyl Sulfoxide as a Drug Delivery System
Source: Gels. 2024 Nov 20;10(11):753. doi: 10.3390/gels10110753 (PMC11593573; doi:10.3390/gels10110753)
Supplement: Supplementary file 1 [file gels-10-00753-s001.zip › gels-3282739-supplementary.pdf]

Supplementary information

Development and Characterization of PVA-Based Organogels for Sustained Drug Release and Antimicrobial activity

Otarbayeva Sabina[1 ʃ] and Berillo Dmitriy [1,2 ʃ*]

[1] Department of Chemistry and Biochemical Engineering, Satbayev University, Almaty 050013, Kazakhstan;

[2] Department Biochemistry, Asfendiyarov Kazakh National Medical University, Almaty 050000, Kazakhstan

ʃ Authors contributed equally

* Correspondence: berillo.d@kaznmu.kz

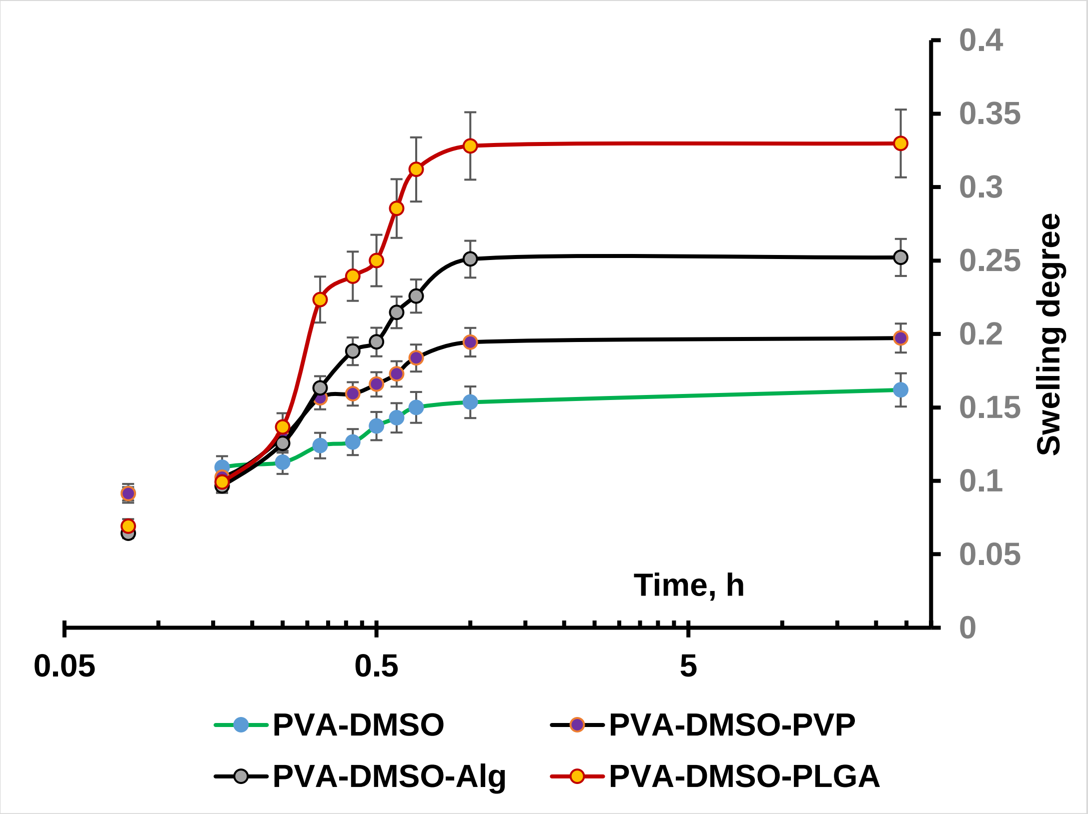

Figure S1 – Swelling curve of PVA organogels in carbonate buffer pH 7.4

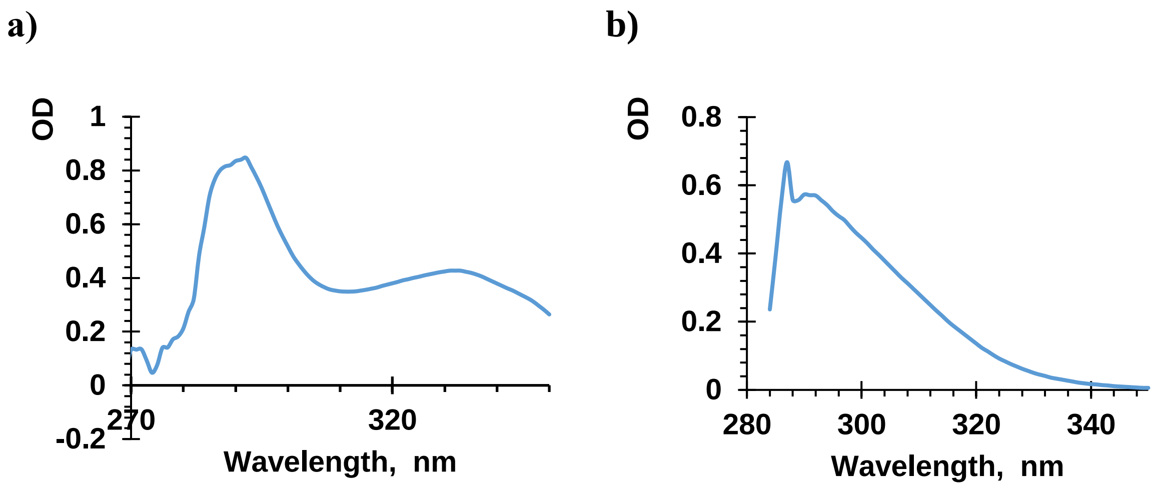

Figure S2 – UV spectra of aqueous solution of a) *ofloxacin*; b) *ceftriaxone*

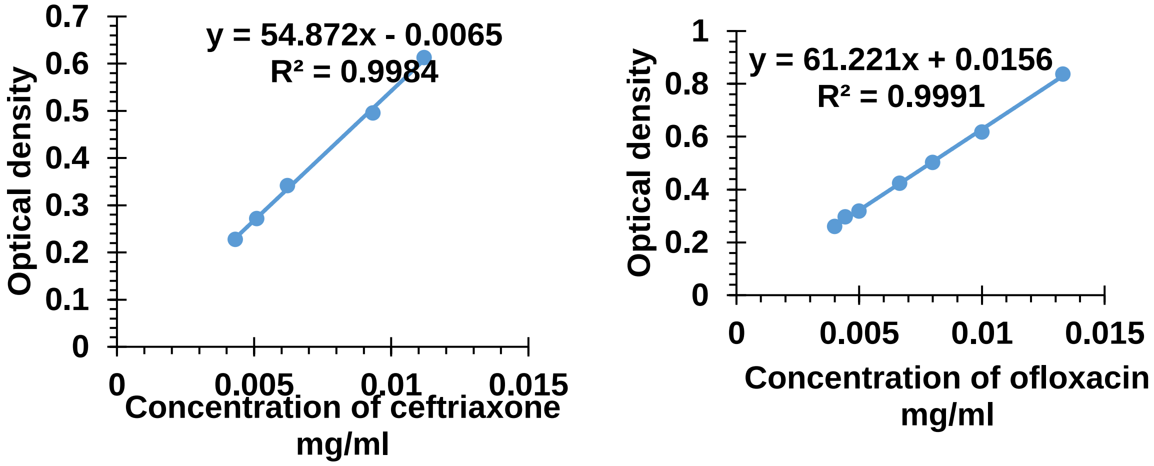

Figure S3 – Calibration curve for *ceftriaxone at* 290 nm and *ofloxacin* at 293nm (SHIMADZU 6000i)

Table S1– A relative visual transparency level of organogels 10% PVA without and with drugs ofloxacin and ceftriaxone.

	PVA-DMSO	PVA-PVP(1%)-DMSO	PVA-Alg(1%)-DMSO	PVA-PLGA(4%)-DMSO
**Without substance**	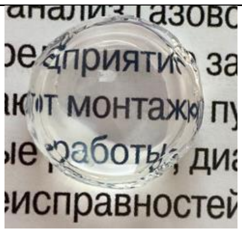	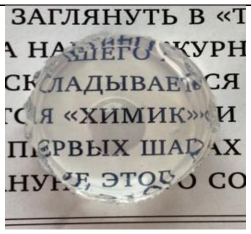	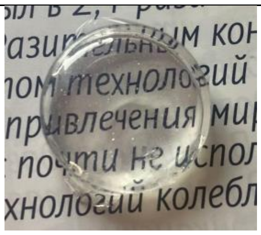	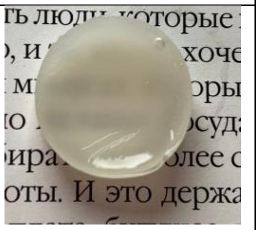

**Oflox**	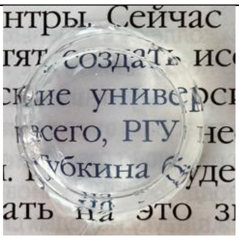	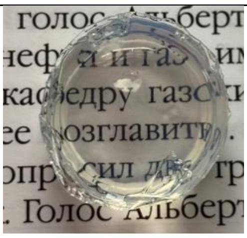	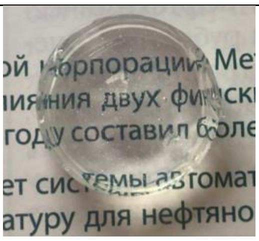	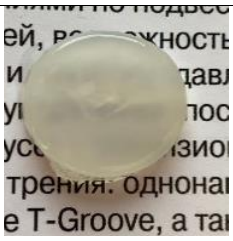
**CEF**	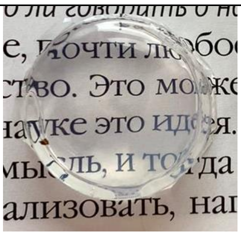	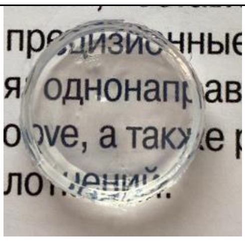	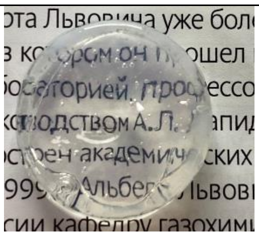	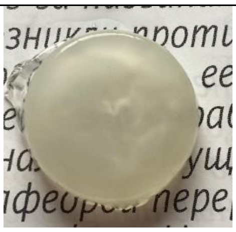
